# Supplementary material for: An open-source framework for large-scale, flexible evaluation of biomedical text mining systems
Source: J Biomed Discov Collab. 2008 Jan 29;3:1. doi: 10.1186/1747-5333-3-1 (PMC2276192; doi:10.1186/1747-5333-3-1)
Supplement: Additional file 1 — Evaluation platform source code. The additional file contains the software evaluation framework discussed in this paper. The most current release can be downloaded from SourceForge.net [1]. See the accompanying README file for instructions on installation and use. Also included in the distribution is a collection of UIMA wrappers for some commonly used BioNLP tools and annotated corpora. [file 1747-5333-3-1-S1.zip › bionlp_uima_repository-1.0.1/docs/api/edu/uchsc/ccp/uima/cc/summary/comparison/AnnotationGroupID.html]

AnnotationGroupID (Center for Computational Pharmacology Util/UIMA API)


|  |  |  |
| --- | --- | --- |
| **Overview** \| **Package** \| **Class** \| **Tree** \| **Deprecated** \| **Index** \| **Help** | |  |
| **PREV CLASS**   **NEXT CLASS** | **FRAMES**    **NO FRAMES**     **All Classes** |  |
| SUMMARY: NESTED \| FIELD \| CONSTR \| METHOD | DETAIL: FIELD \| CONSTR \| METHOD |  |


---


## edu.uchsc.ccp.uima.cc.summary.comparison Class AnnotationGroupID

```
java.lang.Object
  edu.uchsc.ccp.uima.cc.summary.comparison.AnnotationGroupID
```

**All Implemented Interfaces:**: Comparable

---

``` public class AnnotationGroupID extends Object implements Comparable ```

---

**Constructor Summary**

`AnnotationGroupID()`

`AnnotationGroupID(String id)`


| **Method Summary** | |
| --- | --- |
| `int` | `compareTo(Object o)` |
| `boolean` | `equals(Object obj)` |
| `String` | `getId()` |
| `int` | `hashCode()` |
| `void` | `setId(String id)` |
| `String` | `toString()` |

**Methods inherited from class java.lang.Object**

`clone, finalize, getClass, notify, notifyAll, wait, wait, wait`

**Constructor Detail**

### AnnotationGroupID

```
public AnnotationGroupID()
```

---


### AnnotationGroupID

```
public AnnotationGroupID(String id)
```


**Method Detail**

### getId

```
public String getId()
```

---


### setId

```
public void setId(String id)
```

---


### toString

```
public String toString()
```

:   **Overrides:**: `toString` in class `Object`

---


### compareTo

```
public int compareTo(Object o)
```

:   **Specified by:**: `compareTo` in interface `Comparable`

---


### equals

```
public boolean equals(Object obj)
```

:   **Overrides:**: `equals` in class `Object`

---


### hashCode

```
public int hashCode()
```

:   **Overrides:**: `hashCode` in class `Object`


---


|  |  |  |
| --- | --- | --- |
| **Overview** \| **Package** \| **Class** \| **Tree** \| **Deprecated** \| **Index** \| **Help** | |  |
| **PREV CLASS**   **NEXT CLASS** | **FRAMES**    **NO FRAMES**     **All Classes** |  |
| SUMMARY: NESTED \| FIELD \| CONSTR \| METHOD | DETAIL: FIELD \| CONSTR \| METHOD |  |


---
